# Research on the Springback Behavior of 316LN Stainless Steel in Micro-Scale Bending Processes

**DOI:** 10.3390/ma15186373

**Published:** 2022-09-14

**Authors:** Shubiao Guo, Chenchen Tian, Haitao Pan, Xuefeng Tang, Lu Han, Jilai Wang

**Affiliations:** 1 Key Laboratory of High Efficiency and Clean Mechanical Manufacture of Ministry of Education, School of Mechanical Engineering, Shandong University, Jinan 250061, China; 2 National Demonstration Center for Experimental Mechanical Engineering Education, Shandong University, Jinan 250061, China; 3 State Key Laboratory of Materials Processing and Die & Mould Technology, School of Materials Science and Engineering, Huazhong University of Science and Technology, Wuhan 430074, China; 4 Rizhao Intelligent Manufacturing Institute, Shandong University, Rizhao 276800, China

**Keywords:** micro-bending, size effect, springback, strain gradient, constitutive model

## Abstract

Hydrogen fuel cells have been used worldwide due to their high energy density and zero emissions. The metallic bipolar plate is the crucial component and has a significant effect on a cell’s efficiency. However, the springback behavior of the metallic bipolar plate will greatly influence its forming accuracy in the micro-scale sheet metal forming process. Therefore, accurate calculation of the springback angle of the micro-scale metallic bipolar plate is urgent but difficult given the state of existing elastoplastic theory. In this paper, a constitutive model that simultaneously considers grain size effect and strain gradient is proposed to analyze micro-scale bending behavior and calculate springback angles. The specialized micro-scale four-point bending tool was designed to better calculate the springback angle and simplify the calculation step. A pure micro-bending experiment on a 316LN stainless steel sheet with a thickness of 0.1 mm was conducted to verify the constitutive model’s accuracy.

## 1. Introduction

Hydrogen fuel cells have drawn considerable attentions due to their high power density, high efficiency and zero emissions [1,2]. The metallic bipolar plate is the crucial component of the hydrogen fuel cell and has a significant effect on a cell’s efficiency. The fabrication method of the bipolar plate includes chemical corrosion [3], hydroforming and mechanical bonding [4], soft film forming [5,6] and so on. The thickness of the micro-channel in the metallic plate, which belongs to the micro-forming field, is smaller than 1 mm. The micro-forming refers to the forming process of the part with at least two dimensions in the sub-millimeter range [7]. Currently, silica-based and non-silica-based micro-nano-machining methods are widely used in micro-scale part forming [8,9]. Springback behavior can greatly influence the dimensional accuracy of the deformed part in the micro-scale sheet metal forming process [10,11]. Thus, it is worth seeking to understand springback behavior in metallic micro-scale forming in order to enhance forming accuracy and reduce the number of parts wasted due to springback.

In the micro-scale sheet metal forming process, the main factors affecting springback behavior include the grain size effect [8,12] and the strain gradient [13]. Size effect influences the deformation behavior significantly in the micro-scale forming process [14,15], which is mainly divided into two kinds: ‘the smaller the weaker’ [16] and ‘the smaller the stronger’ [17]. Diehl et al. [18] conducted bending tests using Cu58 sheets with thicknesses ranging from 25 to 500 μm and proved that the ratio of grain size to sheet thickness exerts an influence on the springback angle. Li et al. [19] also found that the springback angle of pure aluminum will increase as metal sheet thickness decreases during the micro-bending test and proposed an analytical model considering the size effect to calculate the springback angle. Gau et al. [20] conducted a three-point bending experiment which indicated that the springback angle can be expressed as a function of the ratio of grain size to sheet thickness when the thickness is less than 350 μm. Xu et al. [11] analyzed the result of the V-bending experiment and indicated that springback behavior is affected by geometry and grain size, and proposed a finite element model based on the surface layer model to analyze the springback angle and describe the size effect of springback behavior.

The dislocation slip theory is the basic theory for the metal plastic forming process and the dislocations are mainly divided into two kinds: statistically stored dislocations (SSDs) and geometrically necessary dislocations (GNDs) [21]. SSDs play the leading roles in uniform deformation [22], while GNDs play the leading roles in non-uniform deformation, where a strain gradient exists [23]. A strain gradient exists in the micro-bending process due to the leading role of GNDs. Diehl et al. [18] mentioned the contribution of strain gradients and size effects in metal sheet micro-forming processes where sheet thicknesses are small. Li et al. [22] proposed a model considering both plastic strain and plastic strain gradients to predict springback angle, indicating the influence of the strain gradient hardening effect. To better explain the relationship between size effect and strain gradient, Wang et al. [24] proposed a constitutive model considering both size effects and strain gradients to investigate the interactions between them.

However, it is difficult to calculate the springback angle of a metallic sheet in the micro-bending process based on the analytical model due to the limitations of forming tooling. For example, the bending area is hard to confirm for U-bending tooling [25] and the bending moment is variable for three-point bending tooling [26]. To solve these problems, Deng et al. [27] designed a four-point bending tooling to obtain the pure bending moment with a fixed value, which is beneficial for precisely calculating the springback angle.

Based on the research mentioned above, springback behavior is affected by the size effect and the strain gradient simultaneously, but their relationship and their influence on springback angle have not been fully understood. To better understand the mechanism of springback behavior, a constitutive model considering both size effect and strain gradient is proposed in this paper. This model is used to explain size effects based on a surface layer model with a high order of strain. To enhance the model’s accuracy and simplify the calculation procedure, a specialized micro-scale four-point bending tooling was designed. The proposed model which simultaneously considers size effect and strain gradient is experimentally validated.

## 2. Micro-Scale Four-Point Bending Experiment

### 2.1. Material Preparation

**Figure 1:**
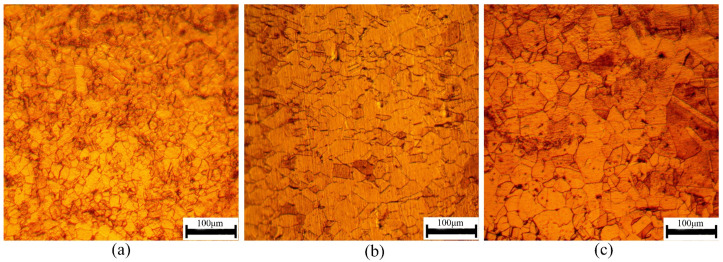
Microstructures of the 316LN stainless steel annealed at (**a**) 900 °C, (**b**) 950 °C and (**c**) 1000 °C.

Stainless steel is the main material used to make metallic bipolar plates due to its good corrosion resistance and formability. In this study, 316LN stainless steel with a Young’s modulus of E = 50–60 GPa, a Poisson’s ratio of ν = 0.29 and a density of ρ = 7.98 × 10^3^ kg/m^3^ was selected to assess size effect and strain gradient size. Three annealing treatments were carried out to eliminate the effect of rolling texture and obtain different grain sizes. To explore the influence of grain size on mechanical behavior in the micro-bending process, the specimen thickness was fixed as 0.1 mm. The heat-treatment conditions and obtained grain sizes are presented in Table 1. The samples were corroded with aqua regia (volume fraction ratio = HCl (37%):HNO_3_ (68%) = 3:1) for 50 s to obtain the microstructure micrographs shown in Figure 1. The specimens after annealing treatment were isotropic. The plane we used for recording was perpendicular to the thickness direction. The micrographs were observed using a metallurgical microscope (HYZX-2000, Laizhou, China).

### 2.2. Tensile Tests

**Figure 2:**
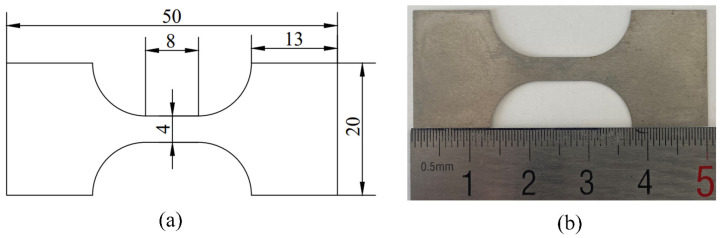
Tensile test specimens: (**a**) designed specimen; (**b**) machined specimen.

To determine the mechanical properties of the stainless steel, uniaxial tensile tests were conducted in the MTS testing machine. The specimens with different grain sizes were tested three times to ensure testing accuracy. The figure of the tensile specimen was designed in accordance with the ASTM-E8 standard to eliminate stress concentration and is shown in Figure 2. The crosshead velocity was set as 0.033 mm/s, and the strain rate was set as 4.125 × 10^−3^ s^−1^.

**Figure 3:**
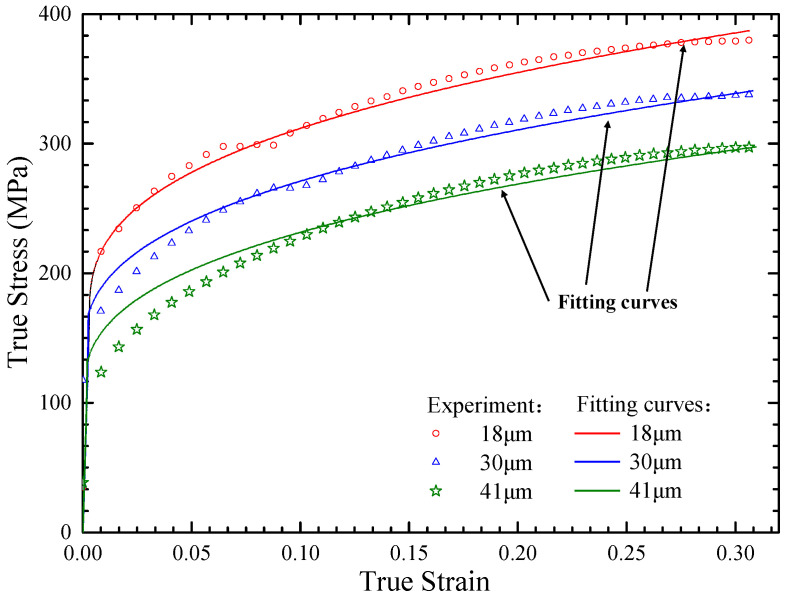
True stress–strain curves for the tensile specimens with thicknesses of 0.1 mm.

The true stress–strain curves for the tensile specimens are presented in Figure 3. In this research, the springback behavior analysis did not need to consider the damage stage where the strain was bigger than 0.3. It can be seen from Figure 3 that the flow stress decreased with the increase in grain size at the same strain and strain rate. The flow stress of the materials with different grain sizes varied significantly. The stress–strain curves had good repeatability and consistency for the three different sheet metal samples.

### 2.3. Micro-Scale Four-Point Bending Experiments

**Figure 4:**
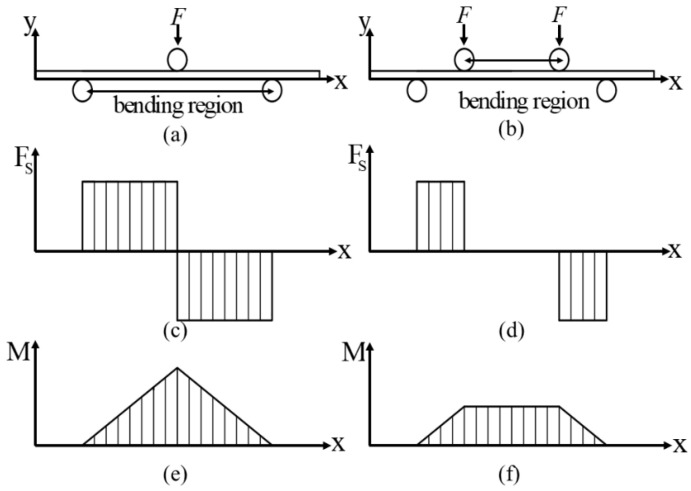
(**a**) Three-point bending diagram. (**b**) Four-point bending diagram. (**c**) Three-point bending shear force diagram. (**d**) Four-point bending shear force diagram. (**e**) Three-point bending moment diagram. (**f**) Four-point bending moment diagram.

**Figure 5:**
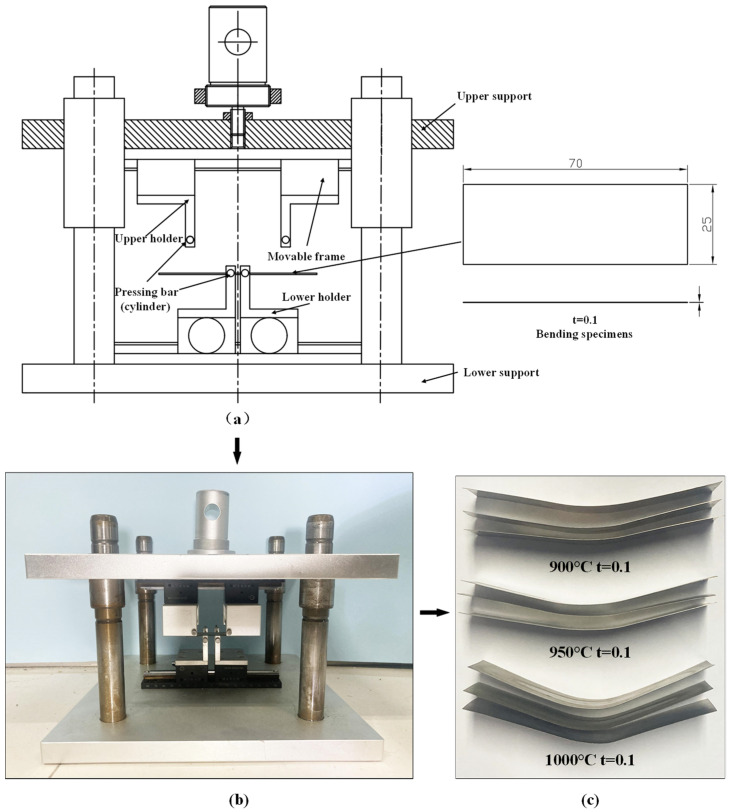
Experimental tool and corresponding specimens: (**a**) the structure of the micro-bending tool; (**b**) a picture of the micro-bending tool; (**c**) deformed specimens.

The calculation of the springback angle of a metal sheet is difficult because the bending moment in the bending area is variable [24]. The principle and force analyses of the three- and four-point bending experiments are shown in Figure 4a,b, respectively. To simplify the calculation step and investigate the springback behavior, the specialized tooling for the four-point bending test was designed to conduct the pure bending deformation experiment shown in Figure 5. The punch and die were designed with the shapes of long cylinders which could freely rotate to reduce friction. To reduce the influence of friction, lubricating oil was applied at the interface. In order to increase the tooling’s flexibility, two movable frames controlled by pinions and racks were applied to the tooling, whose mobile precision could reach 0.1 mm. The specific sizes of the tooling parts are shown in Table 2. The specimens were 70 mm in length and 25 mm in width.

The experimental results for the springback angles of the specimens are shown in Table 3. They reveal that the average springback angle decreases with increments in grain size. That is, the ratio of surface grain size to sheet thickness will increase with the increase in grain size when the thickness is fixed, making it easier to deform plastically for the metal sheet material [28], which corresponds to the “the smaller the stronger” effect.

## 3. A Combined Constitutive Model

From the flow stress–strain curve, the deformation process can be divided into two stages: the elastic deformation stage and the plastic deformation stage [29]. In the elastic deformation stage, Hooke’s law was applied. In the plastic deformation stage, the proposed constitutive model was used to explain the phenomenon. The relation is shown in Equation (1).

(1) $$\sigma \left(\varepsilon \right)=\{\begin{array}{cc} E\varepsilon & 0\leq \varepsilon \leq \varepsilon_{0}\\ \sigma_{0}+\sigma_{size}+\sigma_{gradient} & \varepsilon \geq \varepsilon_{0} \end{array}$$

### 3.1. A Combined Constitutive Model

**Figure 6:**
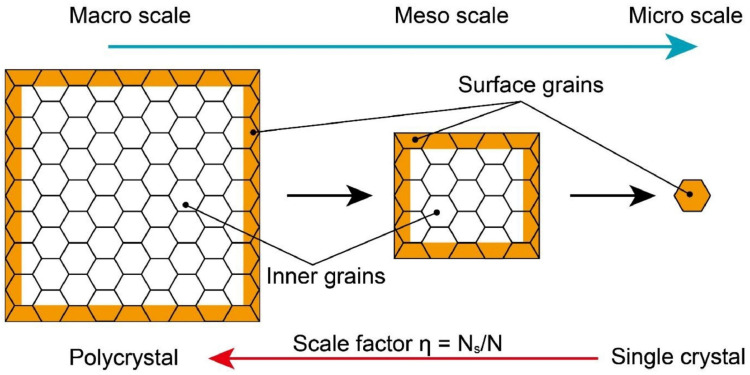
Schematic identification of the surface and inner grains in a workpiece as a function of overall scale [30].

The basic theory of the surface layer model divides a sheet into two parts: a surface part and an inner part, both of which are shown in Figure 6. As the two parts play different roles in the sheet, the flow stress of the sheet can also be explained in two parts. The surface part’s grains will undergo less constraint, which may cause easy sliding and rotation. On the contrary, the inner part’s grains will experience more constraint where sliding and rotation are limited. The sheet’s flow stress can be expressed using Equation (2), giving the relationship between the inner and surface grains.

(2) $$\left\{ \begin{array}{l} \sigma =\eta \sigma_{s}+(1-\eta )\sigma_{i}\\ \eta =\frac{N_{s}}{N} \end{array}\right.$$

In Equation (2), $N_{s}$ and N refer to the numbers of surface grains and total grains, respectively; $\sigma_{s}$, $\sigma_{i}$ and σ refer to the flow stresses of the surface part, the inner part and the whole, respectively; η represents the number ratio of surface grains to total grains. When η increases to 1, the specimen can be regarded as a single crystal, and the surface grain exerts a great influence on the deformation process.

The proposed hybrid constitutive model [30] considers the size effect on the basis of surface layer model to describe the relationship between flow stress and strain. The surface grain and inner grain are considered as the single crystal and the polycrystal, respectively, to better describe the property. The stress of surface strain and inner strain can be deduced from the crystal plasticity theory [31] and the Hall–Petch equation [32], which are shown in Equations (3) and (4).

(3) $$\sigma_{s}\left(\varepsilon \right)=m\tau_{0}\left(\varepsilon \right)$$

(4) $$\sigma_{i}\left(\varepsilon \right)=M\left(\tau_{0}\left(\varepsilon \right)+k\left(\varepsilon \right)d^{-\frac{1}{2}}\right)$$

where the factor d represents the grain size; the parameters m and M represent the orientation factors for a single crystal and a polycrystal, respectively; $\tau_{0}\left(\varepsilon \right)$ represents the critical resolved shear stress of a single crystal; and k(ε) represents the local stress needed for general yield associated with the transmission of slip across polycrystal grain boundaries.

According to crystal plasticity theory, using lattice friction stress $\tau_{0}\left(\varepsilon \right)$ and dislocation-introduced hardening to represent shear stress $\tau_{R}\left(\varepsilon \right)$, Equation (5) can be obtained:

(5) $$\tau_{R}\left(\varepsilon \right)=\tau_{0}\left(\varepsilon \right)+\alpha Gb\sqrt{\rho_{T}}=\tau_{0}\left(\varepsilon \right)+\alpha Gb\sqrt{\rho_{S}+\rho_{G}}$$

where α is the empirical constant ranging from 0.1 to 0.5 and b is the Burgers vector magnitude, which in the case of FCC crystal is $2.608\times 10^{-10}\;m$ [33]; G is the shear modulus; and the parameters $\rho_{T},\rho_{S},\rho_{G}$ represent the total dislocation density, SSD and GND, respectively. The latter two are the monotonic functions of strain described in Equations (6) and (7).

(6) $$\rho_{S}\left(\varepsilon \right)=\frac{C_{S}\varepsilon }{bL^{S}}$$

(7) $$\rho_{G}\left(\varepsilon \right)=\frac{C_{G}\varepsilon }{bd}$$

In Equations (6) and (7), $C_{S}$ and $C_{G}$ are the material constants and $L^{S}$ is the slip length [33]. Thus, the total dislocation density can be represented by Equation (8):

(8) $$\rho_{T}=\rho_{S}+\rho_{G}=\frac{C_{S}\varepsilon }{bL^{S}}+\frac{C_{G}\varepsilon }{bd}$$

The SSD exerts more influence towards classic plasticity, which is at the macro-scale; however, when the scale is reduced to the micro-scale, the GND may have a dominant effect on the deformation behavior and perhaps the SSD’s effect can be ignored, as in a situation described by the following expression: $\rho_{S}\left(\varepsilon \right)=0$. Based on Swift’s hardening model, $\tau \left(\varepsilon \right)=k{\left(\varepsilon \right)}^{n}$, the constitutive model of the deformation behavior at the macro-scale can be described using Equation (9):

(9) $$\begin{array}{cc} \sigma_{i} & =M\left(\tau_{0}\left(\varepsilon \right)+\alpha Gb\sqrt{\rho_{T}}\right)=M\left(\tau_{0}\left(\varepsilon \right)+\alpha Gb\sqrt{\frac{C_{G}\varepsilon }{bd}}\right)\\ & =Mk{\left(\varepsilon \right)}^{n}+M\alpha Gb\sqrt{\frac{C_{G}\varepsilon }{bd}} \end{array}$$

To obtain the constitutive model at the micro-scale, Equations (2) and (9) can be combined to obtain Equation (10):

(10) $$\sigma \left(\varepsilon \right)=\eta mk_{1}{\left(\varepsilon \right)}^{n_{1}}+\left(1-\eta \right)\left(Mk_{2}{\left(\varepsilon \right)}^{n_{2}}+M\alpha Gb\sqrt{\frac{C_{G}\varepsilon }{bd}}\right)$$

In Equation (10), the model can represent the polycrystal model in the case of η=0, and when η=1, the model represents the single crystal model as well.

**Figure 7:**
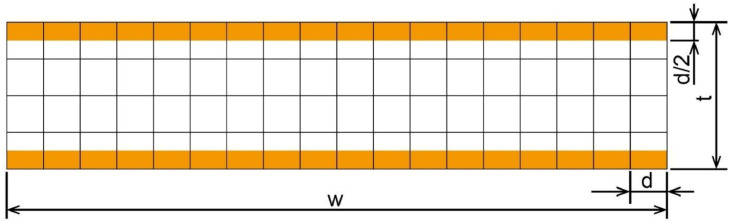
The surface layer model of the sheet samples at the micro-scale [24].

It can be seen from Figure 7 that d represents the diameter of the grain, using t and w to represent the plate’s thickness and width, respectively. It is apparent that the width is much bigger than the thickness and grain size; thus, Equation (10) can be used to simplify the parameter η, as expressed in Equation (11):

(11) $$\eta =\frac{N_{S}}{N}=\frac{2\left(wd/2\right)d^{2}}{wt/d^{2}}=\frac{d}{t}$$

The curve can be used to fit the parameters $k_{1},k_{2},n_{1},n_{2}.$ The results are $C_{G}=0.18,\alpha =0.34$ [34], and m and M [35,36] are set as 2 and 3.06, respectively.

The calculation results and the true stress–strain curve obtained from the experiments can be seen in Figure 3. The solid line represents the fitted result and the marker line represents the experimental result, showing that the fitted curve is in good agreement with the experimental results.

### 3.2. Constitutive Model Considering Strain Gradient

At the micro-scale, the plastic strain gradient hardening exerts a greater influence on deformation than at the macro-scale. To better describe this fact, putting the strain gradient into the constitutive model yields Equation (12):

(12) $$\sigma \left(\varepsilon \right)=\eta mk_{1}{\left(\varepsilon \right)}^{n_{1}}+\left(1-\eta \right)\left(Mk_{2}{\left(\varepsilon \right)}^{n_{2}}+M\alpha Gb\sqrt{\frac{C_{G}\varepsilon }{bd}}\right)+k_{3}l\left|\nabla \varepsilon \right|$$

where |∇ε| is the plastic strain gradient’s contribution to flow stress, l represents the intrinsic length and $k_{3}=k_{1}=k_{2}$.

The gradient (along the thickness direction) of the plastic strain along the longitudinal direction of the sheet is shown in Equation (13):

(13) $$\nabla \varepsilon =\frac{\left[\left(R_{i}+t\right)d\theta -R_{n}d\theta \right]-\left(R_{i}d\theta -R_{n}d\theta \right)}{tR_{n}d\theta }=\frac{1}{R_{n}}=c$$

In Equation (13), parameter c denotes the neutral layer’s curvature; $R_{i}$ and $R_{n}$ are the inner side of the metal sheet’s radius and the radius of the neutral layer, respectively; and θ is the bending area’s angle after bending.

Different materials have different intrinsic lengths, especially when there are differences in hardness. In addition, the same material can have different intrinsic lengths when micro-structures are different. For instance, the intrinsic lengths of the single-crystal copper alloy and the polycrystal copper alloy are different [22].

Based on the relationship of intrinsic length, the material properties insisted on by Xue et al. [37] and Swift’s hardening model, the material intrinsic length can be calculated using Equation (14):

(14) $$l=18\alpha^{2}{\left(\frac{G}{\sigma_{S0}}\right)}^{2}b$$

### 3.3. The Calculation of Strain, Strain Gradient, Stress and Bending Moment

#### 3.3.1. The Calculation of Strain and Strain Gradient

**Figure 8:**
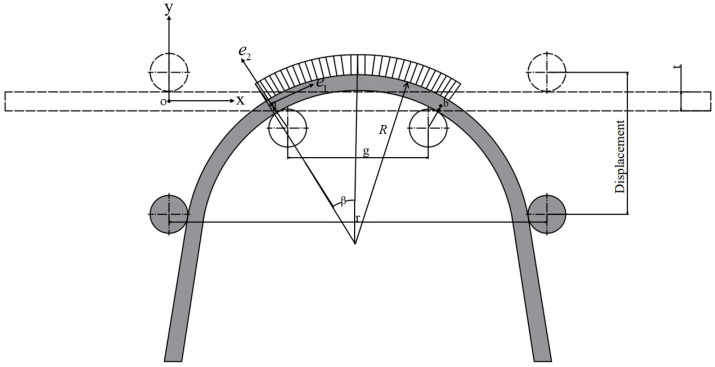
Geometrical schematic diagram of micro-bending deformation.

The geometrical model of micro-bending deformation is shown in Figure 8. ${\vec{e}}_{1},{\vec{e}}_{2},{\vec{e}}_{3}$ are the unit vectors along the length, thickness and width directions, respectively. As shown in Figure 4d,f, the bending area undergoes a uniform bending moment and zero shear force, indicating that only normal stress exists along the length direction in the cross section. Therefore, the displacement field is supposed to be as expressed in Equation (15):

(15) $$\mu_{1}=ce_{1}e_{2},\mu_{2}=0,\mu_{3}=0$$

The strain tensor can be expressed using Equation (16):

(16) $$\left[\varepsilon_{ij}\right]=\left[\begin{array}{ccc} ce_{2} & 0 & 0\\ 0 & 0 & 0\\ 0 & 0 & 0 \end{array}\right]$$

For the deformation behavior seen in a uniaxial test, the strain is shown in Equation (17):

(17) $$\varepsilon_{1}=ce_{2}$$

The gradient of strain is represented by Equations (18) and (19):

(18) $$\nabla \varepsilon =\left[\begin{array}{ccc} 0 & 1 & 0 \end{array}\right]$$

(19) |∇ε|=c

Putting Equations (17) and (19) into Equation (12), the constitutive relationship of the analytical model can be expressed using Equation (20):

(20) $$\bar{\sigma }\left(\varepsilon \right)=\eta mk_{1}{\left(ce_{2}\right)}^{n_{1}}+\left(1-\eta \right)\left[Mk_{2}{\left(ce_{2}\right)}^{n_{2}}+M\alpha Gb\sqrt{\frac{C_{G}}{bd}}\sqrt{ce_{2}}\right]+k_{3}lc$$

#### 3.3.2. The Calculation of Stress

In the pure bending district, deformation behavior can be considered in the uniaxial tensile test; thus, the normal stress only exists in the length direction. Therefore, the stress is as shown in Equation (21):

(21) $$\sigma_{2}=0,\sigma_{3}=0$$

Thus, the effective stress is shown in Equation (22):

(22) $$\bar{\sigma }=\sqrt{\frac{3}{2}\sigma_{ij}^{\prime }\sigma_{ij}^{\prime }}=\sigma_{1}$$

During micro-bending deformation, the metal sheet can deform elastically and plastically, and in the district where it deforms elastically, the stress is shown in Equation (23):

(23) $$\sigma =E\varepsilon \;0\leq \varepsilon \leq \varepsilon_{0}$$

where $\varepsilon_{E}$ is the elastic strain limit.

To obtain the plastic deformation district’s constitutive equation by substituting Equation (22) into Equation (20), the formula can be expressed as Equation (24):

(24) $$\begin{array}{cc} \sigma_{1} & =\eta mk_{1}{\left(ce_{2}-\varepsilon_{0}\right)}^{n_{1}}\\ & +\left(1-\eta \right)\left[Mk_{2}{\left(ce_{2}-\varepsilon_{0}\right)}^{n_{2}}+M\alpha Gb\sqrt{\frac{C_{G}}{bd}}\sqrt{ce_{2}-\varepsilon_{0}}\right]+k_{3}lc \end{array}$$

In total, the constitutive model can be derived as Equation (25):

(25) $$\sigma \left(\varepsilon \right)=\{\begin{array}{c} E\varepsilon \\ \left\{ \begin{array}{cc} \sigma_{0}+\eta mk_{1}{\left(\varepsilon -\varepsilon_{0}\right)}^{n_{1}} & 0\leq \varepsilon \leq \varepsilon_{0}\\ +\left(1-\eta \right)\left(Mk_{2}{\left(\varepsilon -\varepsilon_{0}\right)}^{n_{2}}+M\alpha Gb\sqrt{\frac{C_{G}\left(\varepsilon -\varepsilon_{0}\right)}{bd}}\right)+k_{3}l\left|\nabla \varepsilon \right| & \varepsilon \geq \varepsilon_{0} \end{array}\right. \end{array}$$

#### 3.3.3. The Calculation of Bending Moment

To calculate the bending moment, use Equation (26).

(26) $$M=\int_{0}^{t}\sigma_{1}e_{2}w\cdot de_{2}$$

**Figure 9:**
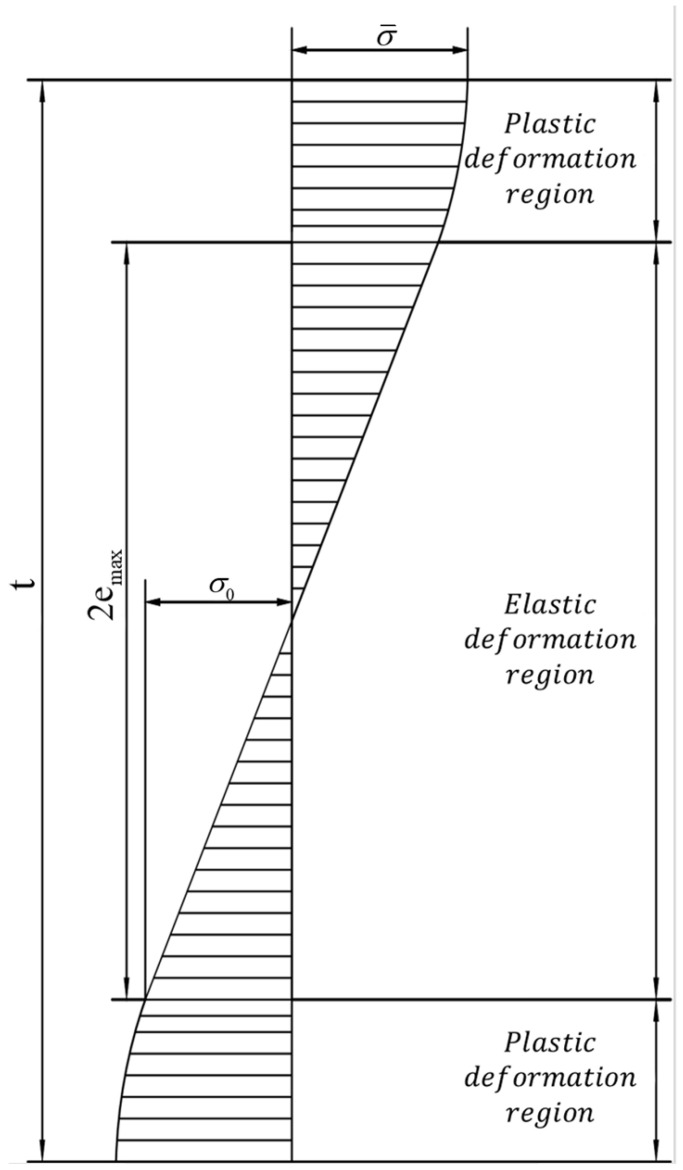
Stress distribution along sheet thickness direction.

For the stress in the elastic district as in Figure 9, based on Equations (23) and (26), the elastic bending moment can be expressed using Equation (27):

(27) $$M_{E}=\int_{-e_{\max}}^{e_{\max}}\sigma_{1}e_{2}w\cdot de_{2}=\frac{2wE}{3}c{\left(e_{\max}\right)}^{3}$$

For the stress in the elastic district as in Figure 9, based on Equations (24) and (26), the plastic bending moment can be expressed as Equation (28):

(28) $$\begin{array}{cc} M_{P} & =2\int_{e_{\max}}^{t/2}\sigma_{1}e_{2}w\cdot de_{2}\\ & =2w\int_{e_{\max}}^{t/2}\{\sigma_{0}+\eta mk_{1}{\left(ce_{2}-ce_{\max}\right)}^{n_{1}}+\left(1-\eta \right)[Mk_{2}{\left(ce_{2}-ce_{\max}\right)}^{n_{2}}\\ & +M\alpha Gb\sqrt{\frac{C_{G}}{bd}}\sqrt{ce_{2}-ce_{\max}}]+k_{3}lc\}e_{2}\cdot de_{2}\\ & =2w\left\{\begin{array}{c} \frac{\sigma_{0}}{2}\left[{\left(\frac{t}{2}\right)}^{2}-e_{\max}{}^{2}\right]+\eta mk_{1}{\left(c\right)}^{n_{1}}\left[\frac{{\left(\frac{t}{2}-e_{\max}\right)}^{n_{1}+2}}{n_{1}+2}+e_{\max}\frac{{\left(\frac{t}{2}-e_{\max}\right)}^{n_{1}+1}}{n_{1}+1}\right]\\ +\left(1-\eta \right)Mk_{2}{\left(c\right)}^{n_{2}}\left[\frac{{\left(\frac{t}{2}-e_{\max}\right)}^{n_{2}+2}}{n_{2}+2}+e_{\max}\frac{{\left(\frac{t}{2}-e_{\max}\right)}^{n_{2}+1}}{n_{2}+1}\right]\\ +\left(1-\eta \right)M\alpha Gb\sqrt{\frac{C_{G}}{bd}}\sqrt{c}\frac{2}{5}\left[\frac{{\left(\frac{t}{2}-e_{\max}\right)}^{\frac{5}{2}}}{2.5}+e_{\max}\frac{{\left(\frac{t}{2}-e_{\max}\right)}^{\frac{3}{2}}}{1.5}\right]+\frac{k_{3}lc}{2}\left[{\left(\frac{t}{2}\right)}^{2}-e_{\max}{}^{2}\right] \end{array}\right\} \end{array}$$

For the pure bending district, the plastic bending moment can be written as Equation (29):

(29) $$=2w\left\{\begin{array}{c} \frac{\sigma_{0}}{2}\left[{\left(\frac{t}{2}\right)}^{2}\right]+\eta mk_{1}{\left(c\right)}^{n_{1}}\left[\frac{{\left(\frac{t}{2}-e_{\max}\right)}^{n_{1}+2}-{\left(-e_{\max}\right)}^{n_{1}+2}}{n_{1}+2}+e_{\max}\frac{{\left(\frac{t}{2}-e_{\max}\right)}^{n_{1}+1}-{\left(-e_{\max}\right)}^{n_{1}+1}}{n_{1}+1}\right]\\ +\left(1-\eta \right)Mk_{2}{\left(c\right)}^{n_{2}}\left[\frac{{\left(\frac{t}{2}-e_{\max}\right)}^{n_{2}+2}-{\left(-e_{\max}\right)}^{n_{2}+2}}{n_{2}+2}+e_{\max}\frac{{\left(\frac{t}{2}-e_{\max}\right)}^{n_{2}+1}-{\left(-e_{\max}\right)}^{n_{2}+1}}{n_{2}+1}\right]\\ +\left(1-\eta \right)M\alpha Gb\sqrt{\frac{C_{G}}{bd}}\sqrt{c}\frac{2}{5}\left[\frac{{\left(\frac{t}{2}-e_{\max}\right)}^{\frac{5}{2}}-{\left(-e_{\max}\right)}^{\frac{5}{2}}}{2.5}+e_{\max}\frac{{\left(\frac{t}{2}-e_{\max}\right)}^{\frac{3}{2}}-{\left(-e_{\max}\right)}^{\frac{3}{2}}}{1.5}\right]+\frac{k_{3}lc}{2}\left[{\left(\frac{t}{2}\right)}^{2}\right] \end{array}\right\}$$

#### 3.3.4. The Calculation of Springback Angle

**Figure 10:**
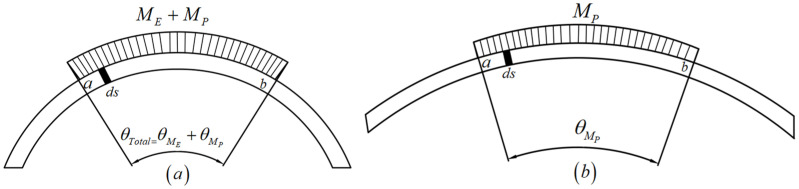
Schematic diagram of a sheet (**a**) after bending and before springback and (**b**) after springback.

The bending angle during the micro-bending process is mainly the result of two factors: the elastic bending moment and the plastic bending moment, both of which are as shown in Figure 10. After the springback process, the angle caused by the elastic bending moment $\left(M_{E}\right)$ disappears, while the angle induced by the plastic bending moment $\left(M_{P}\right)$ continues to exist. Therefore, by calculating the angle $\left(\theta_{M_{P}}\right)$ caused by $M_{P}$, the springback angle $\left(\theta_{S}\right)$ can be calculated indirectly.

After calculating the stress and strain conditions of the micro-bending test, the stress condition of the metal sheet is as described in Equation (28); thus, Equation (28) can be used to calculate the bending moment.

To calculate the springback angle by calculating the angle after springback caused by the plastic bending moment, first, one needs to obtain the infinite small part’s plastic bending angle using Equation (30):

(30) $$d\theta_{M_{P}}=\frac{M_{P}}{IE}\cdot ds$$

where $M_{P}$ represents the segment bending moment in the sheet section, $I=wt^{3}/12$ is the second moment of the area and ds is the segment length, which is illustrated in Figure 10.

Therefore, the total angle caused by the plastic bending moment of the pure bending area can be calculated using Equation (31):

(31) $$\theta_{M_{P}}=\int_{a}^{b}\frac{M_{P}}{IE}\cdot ds=\frac{M_{P}}{IE}\left({\overset{⌢}{S}}_{ab}\right)$$

After bending, ${\overset{⌢}{S}}_{ab}$ and $\theta_{Total}$, caused by the elastic bending moment and the plastic bending moment together, can be calculated. The springback angle can be obtained with Equations (32) and (33):

(32) $${\overset{⌢}{S}}_{ab}=2R\cdot \sin\beta$$

(33) $$\theta_{S}=\theta_{total}-\theta_{M_{P}}$$

## 4. Results and Discussion

### 4.1. Prediction of the Springback Angle

**Figure 11:**
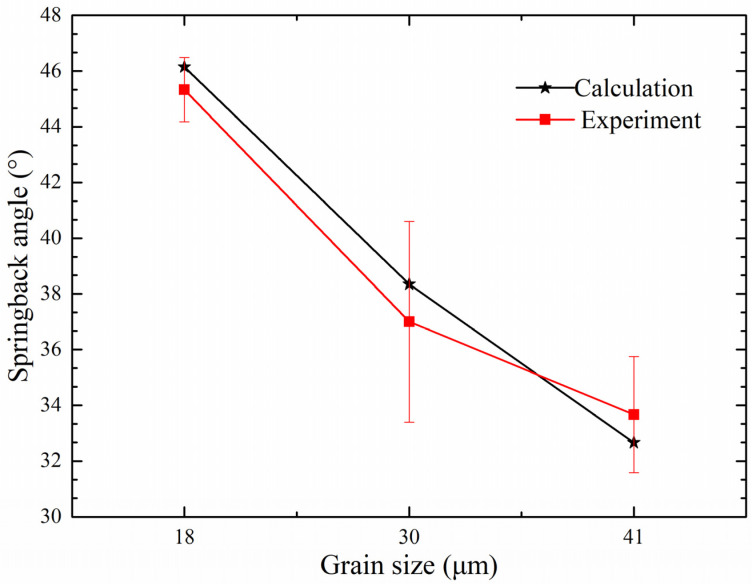
Springback angle of the bending specimen with a thickness of 0.1 mm.

The proposed analytical calculation model can be used to obtain the springback angle. The effects of both grain size and strain gradient on springback angle have been considered and investigated. In addition, there are also many other factors that can influence the springback angle, including the Young’s modulus (E), the sheet thickness (t), the tooling upper bar span (g), the tooling lower bar span (r) and the elastic bending moment (M_E_). In Figure 1a,b, the adopted annealing temperatures are 900 °C and 950 °C, respectively, which do not reach the recrystallization temperature. Therefore, there are few twins in the specimen and the twin effect on the springback angle can be ignored in this research. After conducting the micro-bending tests, the experimental springback angles of the specimens with different grain sizes were compared with the calculation results obtained from the analytical model, which are depicted in Figure 11.

According to Figure 11, it is obvious that the springback angle calculated by the analytical model shows a great similarity with the experimental result. This means that the proposed constitutive model is effective and that it does describe the size effect and the strain gradient accurately. Furthermore, during the plastic deformation, the dislocation is the main reason the surface grain cannot store and pass the dislocation. The surface grain suffers less constraint, thus making it easier to deform the surface area. In conclusion, when the bending angle and the sheet thickness are the same, the θ caused by plastic deformation will increase and the springback angle will decrease with increasing grain size.

### 4.2. Factors Contributing to Springback

**Figure 12:**
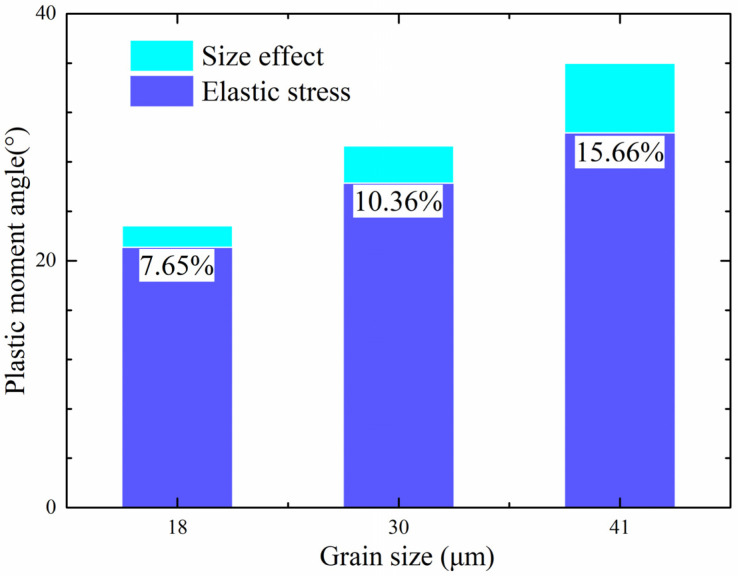
Comparison of the contributions of strain gradient and grain and feature size to springback angles in sheet metal samples with different grain sizes.

The results of the proposed constitutive model, which considers size effect and strain gradient simultaneously, showed great similarity to the experimental data. Therefore, by using the analytical model, the size effect and the strain gradient can be calculated separately, which makes it easy to compare them and investigate their interaction. The contributions of each factor to springback angle are illustrated in Figure 12. It can be seen easily that the size effect makes a greater contribution (more than 7%) than the $\theta_{MP}$ (total angle caused by the plastic bending moment). After the calculations, the strain gradient’s contribution was found to be less than 1% because the 316LN stainless steel mainly suffered elastic deformation rather than plastic deformation during the micro-bending process. Thus, the strain gradient’s contribution was insignificant because the plastic deformation region was small. The size effect’s contribution to the overall springback angle was significant for most cases, in contrast to that of the strain gradient; thus, the size effect exerts a dominant influence on the springback angle, such that the strain gradient’s contribution can be ignored.

**Figure 13:**
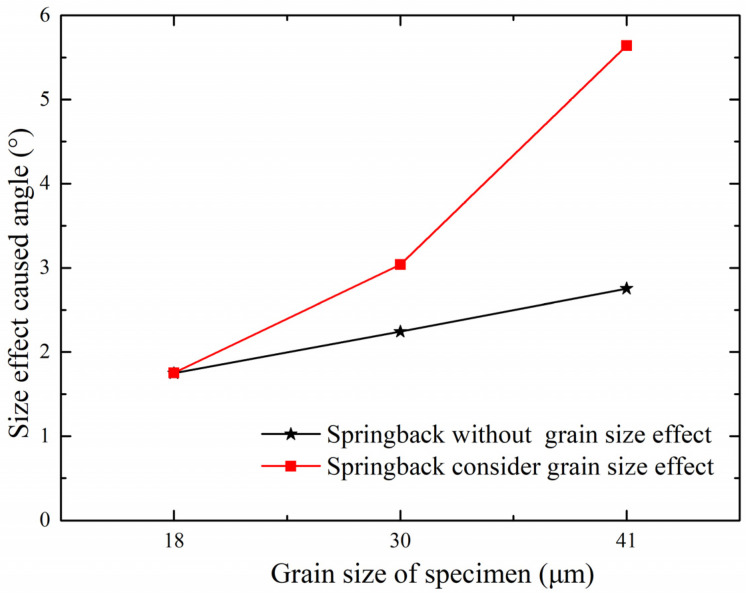
The contribution of strain gradient to springback in sheet metal samples with different grain sizes.

In addition, the contribution of the size effect to the springback will increase with increasing grain size, as shown in Figure 12. It can be seen that the elastic stress’s contribution will decrease while the size effect’s contribution will increase as the grain size increases. The $\theta_{MP}$ caused by the size effect will increase as well. To further investigate the relationship between $\theta_{MP}$ and size effect, the relationship between size-effect-caused angle and grain size is shown in Figure 13. Referring to the black line in Figure 13, if the size effect’s contribution to the plastic bending moment angle stays the same when the grain size equals 18 μm, this indicates that the size effect can be ignored; the red line represents the experimental result that the size effect’s contribution increases with increasing grain size. The difference between these two lines means that the size effect does change with changes in grain size, proving the size effect’s contribution increases as the grain size increases.

## 5. Conclusions

The springback behavior of a metal sheet in the micro-bending process is the result of two main factors, both of which exert influences on the plastic deformation angle and thus influence the springback angle. To further investigate the relationship between these two factors, a uniaxial tensile test was conducted first to obtain stress–strain relationship information. Then, a four-point bending experiment was conducted using a specially designed model with a sheet thickness of 0.1 mm to simplify the calculation process. After that, the proposed model was used, which considers both size effect and strain gradient to predict the springback angle of the micro-bending test. The conclusions drawn from the research are as follows:

1. The springback angle of the micro-bending test shows a ‘the smaller, the stronger’ effect, and the springback angle results calculated using the proposed mixed model which considers size effect and strain gradient showed good agreement with the micro-bending experiment data.
2. The specially designed four-point bending tooling which allowed the obtainment of a pure bending moment in the bending region made the calculation process easier and ensured that the results were accurate.
3. The strain gradient’s effect can be ignored during the micro-bending test that was performed in this study, for the elastic stage of 316LN stainless steel is too obvious, which makes the plastic region small and the strain gradient’s contribution useless, from which it can be inferred that the strain gradient contributes less to materials with obvious elastic stages.
4. Quantitative expressions of the factors in the mixed model can be obtained and compared. The geometrical size effect shows a dominant effect compared to the strain gradient, and its contribution to plastic bending angle increases with increasing grain size.

## Figures and Tables

**Table 1:** Heat-treatment parameters and the corresponding grain sizes.

Annealing Conditions	900 °C, 0.25 h	950 °C, 0.5 h	1000 °C, 1 h
Grain size average (μm)	18.18	29.59	40.51
Grain size deviation (μm)	4.54	8.86	17.33
Grain size/sheet thickness (d/t)	0.18	0.30	0.41

**Table 2:** The size of the tooling.

Bar Radius	Pressing Speed	Upper Bar Span (g)	Lower Bar Span (r)	Pressing Distance
1.25 mm	5 mm/min	15 mm	40 mm	15 mm

**Table 3:** The springback angles of the specimens.

Grain Size (μm)	No.1 (°)	No.2 (°)	No.3 (°)	Average Angle (°)
18	46	44	46	45.3
30	38	33	40	37.0
41	32	33	36	33.7

## Data Availability

The raw/processed data required to reproduce these findings cannot be shared at this time as the data also forms part of an ongoing study.

## References

[1] Tawfik H., Hung Y., Mahajan D. (2007). Metal bipolar plates for PEM fuel cell—A review. J. Power Sources.

[2] Zhang C., Chen S., Wang J., Shi Z., Du L. (2022). Reproducible Flexible SERS Substrates Inspired by Bionic Micro-Nano Hierarchical Structures of Rose Petals. Adv. Mater. Interfaces.

[3] Cho E.A., Jeon U.S., Ha H.Y., Hong S.-A., Oh I.-H. (2004). Characteristics of composite bipolar plates for polymer electrolyte membrane fuel cells. J. Power Sources.

[4] Mahabunphachai S. (2008). A Hybrid Hydroforming and Mechanical Bonding Process for Fuel Cell Biopolar Plates. Ph.D. Thesis.

[5] Peng L., Lai X., Yi P., Mai J., Ni J. (2011). Design, optimization, and fabrication of slotted-interdigitated thin metallic bipolar plates for PEM fuel cells. J. Fuel Cell Sci. Technol..

[6] Peng L., Liu D., Hu P., Lai X., Ni J. (2010). Fabrication of metallic bipolar plates for proton exchange membrane fuel cell by flexible forming process-numerical simulations and experiments. J. Fuel Cell Sci. Technol..

[7] Geiger M., Kleiner M., Eckstein R., Tieslera N., Engel U. (2001). Microforming. CIRP Ann..

[8] Fu M.W., Wang J.L., Korsunsky A.M. (2016). A review of geometrical and microstructural size effects in micro-scale deformation processing of metallic alloy components. Int. J. Mach. Tools Manuf..

[9] Jing C., Wang J., Zhang C., Sun Y., Shi Z. (2022). Influence of size effect on the dynamic mechanical properties of OFHC copper at micro-/meso-scales. Int. J. Adv. Manuf. Technol..

[10] Zhao Y., Peng L., Lai X. (2018). Influence of the electric pulse on springback during stretch U-bending of Ti6Al4V titanium alloy sheets. J. Mater. Processing Technol..

[11] Xu Z., Peng L., Bao E. (2018). Size effect affected springback in micro/meso scale bending process: Experiments and numerical modeling. J. Mater. Processing Technol..

[12] Fu M.W., Wang J.L. (2021). Size effects in multi-scale materials processing and manufacturing. Int. J. Mach. Tools Manuf..

[13] Lou J., Shrotriya P., Allameh S., Buchheit T., Soboyejo W.O. (2006). Strain gradient plasticity length scale parameters for LIGA Ni MEMs thin films. Mater. Sci. Eng. A.

[14] Wang J., Li C., Wan Y., Zhang C., Ran J., Fu M.W. (2020). Size effect on the shear damage under low stress triaxiality in micro-scaled plastic deformation of metallic materials. Mater. Des..

[15] Wang J., Xiao Z., Wang X., Sun Y., Sun C. (2022). Ductile fracture behavior in micro-scaled progressive forming of Magnesium-Lithium alloy sheet. Int. J. Adv. Manuf. Technol..

[16] Kals T.A., Eckstein R. (2000). Miniaturization in sheet metal working. J. Mater. Processing Technol..

[17] Hutchinson J., Fleck N. (1997). Strain gradient plasticity. Adv. Appl. Mech..

[18] Diehl A., Engel U., Geiger M. (2010). Influence of microstructure on the mechanical properties and the forming behaviour of very thin metal foils. Int. J. Adv. Manuf. Technol..

[19] Li H., Dong X., Shen Y., Diehl A., Hagenah H., Engel U., Merklein M. (2010). Size effect on springback behavior due to plastic strain gradient hardening in microbending process of pure aluminum foils. Mater. Sci. Eng. A.

[20] Gau J.T., Principe C., Yu M. (2007). Springback behavior of brass in micro sheet forming. J. Mater. Processing Technol..

[21] Arsenlis A., Parks D.M. (1999). Crystallographic aspects of geometrically-necessary and statistically-stored dislocation density. Acta Mater..

[22] Li H., Dong X., Wang Q., Shen Y., Diehl A., Hagenah H., Engel U., Merklein M. (2011). Determination of material intrinsic length and strain gradient hardening in microbending process. Int. J. Solids Struct..

[23] Gao H., Huang Y. (2003). Geometrically necessary dislocation and size-dependent plasticity. Scr. Mater..

[24] Wang J.L., Fu M.W., Shi S.Q., Korsunsky A.M. (2018). Influence of size effect and plastic strain gradient on the springback behaviour of metallic materials in microbending process. Int. J. Mech. Sci..

[25] Zheng Q., Shimizu T., Yang M. (2017). Grain size effect on mechanical behavior of thin pure titanium foils at elevated temperatures. Int. J. Mech. Sci..

[26] Liu J.G., Fu M.W., Lu J., Chan W.L. (2011). Influence of size effect on the springback of sheet metal foils in micro-bending. Comput. Mater. Sci..

[27] Deng Y.J., Peng L.F., Lai X.M., Fu M.W., Lin Z.Q. (2017). Constitutive modeling of size effect on deformation behaviors of amorphous polymers in micro-scaled deformation. Int. J. Plast..

[28] Peng L., Lai X., Lee H.J., Song J., Ni J. (2009). Analysis of micro/mesoscale sheet forming process with uniform size dependent material constitutive model. Mater. Sci. Eng. A.

[29] Peng L., Liu F., Ni J., Lai X. (2007). Size effects in thin sheet metal forming and its elastic–plastic constitutive model. Mater. Des..

[30] Lai X.M., Peng L.F., Hu P., Lan S., Ni J. (2008). Material behavior modelling in micro/meso-scale forming process with considering size/scale effects. Comput. Mater. Sci..

[31] Han C.S., Gao H., Huang Y., Nix W.D., Hutchinson J.W. (2005). Mechanism-based strain gradient crystal plasticity—I. Theory. J. Mech. Phys. Solids.

[32] Armstrong R.W., Codd I., Douthwaite R.M., Petch N.J. (1962). The plastic deformation of polycrystalline aggregates. Philos. Mag. A J. Theor. Exp. Appl. Phys..

[33] Hansen N. (1985). Polycrystalline strengthening. Metall. Trans. A.

[34] Rodrıguez R., Gutierrez I. (2003). Correlation between nanoindentation and tensile properties: Influence of the indentation size effect. Mater. Sci. Eng. A.

[35] Mecking H., Kocks U.F. (1981). Kinetics of flow and strain-hardening. Acta Metall..

[36] Clausen B., Lorentzen T., Leffers T. (1998). Self-consistent modelling of the plastic deformation of fcc polycrystals and its implications for diffraction measurements of internal stresses. Acta Mater..

[37] Xue Z., Huang Y., Li M. (2002). Particle size effect in metallic materials: A study by the theory of mechanism-based strain gradient plasticity. Acta Mater..

